# Lung Transplant Improves Survival and Quality of Life Regardless of Telomere Dysfunction

**DOI:** 10.3389/fmed.2021.695919

**Published:** 2021-07-30

**Authors:** Lurdes Planas-Cerezales, Elena G. Arias-Salgado, Cristina Berastegui, Ana Montes-Worboys, Rafaela González-Montelongo, José. M. Lorenzo-Salazar, Vanesa Vicens-Zygmunt, Marta Garcia-Moyano, Jordi Dorca, Carlos Flores, Rosario Perona, Antonio Román, María Molina-Molina

**Affiliations:** ^1^ILD Multidisciplinary Unit, Hospital Universitari Bellvitge, IDIBELL, Universitat de Barcelona, Hospitalet de Llobregat, Spain; ^2^Biomedical Research Institute CSIC/UAM, IdIPAZ, Madrid, Spain; ^3^Centro de Investigación Biomédica en Red de Enfermedades Raras, Instituto de Salud Carlos III, Madrid, Spain; ^4^Respiratory Department, Institute of Research, Hospital Universitari Vall d'Hebrón, Universitat Autónoma de Barcelona, Barcelona, Spain; ^5^Genomics Division, Instituto Tecnológico y de Energías Renovables, Santa Cruz de Tenerife, Spain; ^6^Respiratory Department, Hospital Universitario Cruces, Barakaldo, Spain; ^7^Research Unit, Hospital Universitario Nuestra Señora de Candelaria, Santa Cruz de Tenerife, Spain; ^8^Instituto de Tecnologías Biomédicas, Universidad de La Laguna, Santa Cruz de Tenerife, Spain; ^9^Centro Investigación Biomédica en Red de Enfermedades Respiratorias, Instituto de Salud Carlos III, Madrid, Spain

**Keywords:** interstitial lung disease, pulmonary fibrosis, genetics, telomere shortening, telomere disorders, lung transplantation

## Abstract

**Introduction:** Fibrotic interstitial lung diseases (ILDs) are the first indication for lung transplantation (LT). Telomere dysfunction has been associated with poor post-transplant outcomes. The aim of the study was to evaluate the morbi-mortality and quality of life in fibrotic ILDs after lung transplant depending on telomere biology.

**Methods:** Fibrotic ILD patients that underwent lung transplant were allocated to two arms; with or without telomere dysfunction at diagnosis based on the telomere length and telomerase related gene mutations revealed by whole-exome sequencing. Post-transplant evaluation included: (1) short and long-term mortality and complications and (2) quality of life.

**Results:** Fifty-five percent of patients that underwent LT carried rare coding mutations in telomerase-related genes. Patients with telomere shortening more frequently needed extracorporeal circulation and presented a higher rate of early post-transplant hematological complications, longer stay in the intensive care unit (ICU), and a higher number of long-term hospital admissions. However, post-transplant 1-year survival was higher than 80% regardless of telomere dysfunction, with improvement in the quality of life and oxygen therapy withdrawal.

**Conclusions:** Post-transplant morbidity is higher in patients with telomere dysfunction and differs according to elapsed time from transplantation. However, lung transplant improves survival and quality of life and the associated complications are manageable.

## Introduction

Fibrotic interstitial lung diseases (ILDs) are comprised of parenchymal lung disorders with progressive, irreversible fibrosis that present a devastating clinical course (1). Idiopathic pulmonary fibrosis (IPF) is the most lethal fibrotic ILD. Although new anti-fibrotic medications slow disease progression, lung transplantation (LT) remains the treatment option that improves lung function and survival in those cases that can benefit from this procedure (2). After several years with an increasing trend, fibrotic ILD is often now reported as the first indication for LT (3).

In previous studies, pulmonary fibrosis has been linked to aging and repair defects, especially telomere attrition (4–14). Rare coding mutations in telomere-maintenance genes (*TERT, TERC, PARN, RTEL1, DKC1, TINF2, NAF1, ACD, NOP10, NHP2*) and telomere shortening have been identified in different fibrotic ILDs, irrespective of family aggregation (4–6, 9, 10, 12, 15, 16). Regardless of gene mutations and ILD diagnoses (17), telomere shortening has been associated with disease progression, reduced survival, and poor LT outcomes (18, 19). Telomeric disease onset frequently appears in young adults; therefore, LT may be necessary in most patients (18). However, some reports have shown a high morbidity and mortality related to LT in fibrotic ILD cases with telomere defects (20–25). Furthermore, increased immunosuppressive drug-related toxicities have also been reported (26, 27), especially during post-transplant prophylaxis regimens (20–22), probably due to the associated T cell immunodeficiency (28). Nevertheless, most studies evaluating the effect of telomere dysfunction in LT lack a control group that allow to differentiate those effects only attributed to this biological defect (20–24).

A systematic assessment of telomere length in the pre-transplant study could be useful for optimizing LT protocol among fibrotic ILD cases. However, limited data exists about the LT in pulmonary fibrosis depending on telomere biology and the different technical interventional approaches. The aim of the study was to compare the morbidity, mortality, and quality of life of fibrotic ILD patients that underwent LT with and without telomere dysfunction.

## Materials and Methods

### Study Population

This prospective observational study included 20 patients from the ILD Unit of Hospital Universitari de Bellvitge (HUB) with any form of fibrotic ILD that underwent LT at the Hospital Universitari Vall d'Hebrón, in Barcelona. The Ethics Committee of HUB approved the study and all patients provided written informed consent before inclusion. A telomere genetic study was performed at the moment of diagnosis. Diagnosis was performed following the international clinical guidelines for ILDs (29–31). Patients were recruited at the time of referral to the LT evaluation, with a post-transplant follow-up period of at least 3 months. Patients were excluded if they did not consent to the telomere genetic test.

Clinical data were prospectively recorded from patient inclusion. Surgical, post-surgical procedure, and management protocols were blinded to the laboratory tests and followed national and international guidelines (32, 33). The Lung Allocation Score (LAS) was used to assess the allocating process of donated lungs. The decision for unilateral vs. bilateral lung transplant was based on the presence of pulmonary hypertension or microbiological colonization. In those cases, a bilateral lung transplant was performed. A standard immunosuppression regimen was initiated after LT and adjusted as tolerated. The prophylaxis for cytomegalovirus (CMV) was initiated after the surgical procedures in all patients, using ganciclovir 3–5 mg/kg/12 h during the Intensive Care Unit (ICU) stay, followed by valganciclovir 900 mg/day when oral intake was tolerated. The treatment was maintained for 12 months in serum-negative pre-transplant cases and 6 months for those serum-positive. The post-transplant evaluation included: (1) type of complications observed during the first 24 h, the first 30 days, and after the first month; and, (2) assessment of the quality of life after 6 months of LT based on an improvement of forced vital capacity (FVC) and health status questionnaires (K-BILD and ATAQ-IPF), oxygen therapy withdrawal and declared independence for daily life activities.

### Telomere Genetic Studies

The telomere genetic study was performed at fibrotic ILD diagnosis and consisted of: (1) a telomere length screening of DNA isolated from mouth epithelial cells (oral swab) using a commercial DNA isolation kit (Isohelix, Cell Projects Ltd.) (34) and further validation based on DNA from peripheral blood; (2) a sequencing analysis of the known telomere related-genes (TRG) described so far as a cause of pulmonary fibrosis. The assessment of telomere dysfunction in donors was unavailable in our study due to the great inherent lung transplant technical difficulties.

### Telomere Length Analysis

The relative telomere length was performed in the Instituto de Investigaciones Biomédicas (CSIC/UAM) and initially assessed by quantitative polymerase chain reaction (qPCR), as previously described (19, 35, 36). Since telomere length changes with age, a Z-score value was obtained to allow the comparisons of the telomere length between individuals of different ages. The Z-score compared the Telomere Shortening T/S ratio value in each individual with the age-matched mean and standard deviation (SD) of the values obtained in the controls. The Z-score below the 25^th^ percentile of a normal distribution was considered as “short telomere.” A severe telomere length reduction was identified when Z-score was below the 10^th^ percentile.

**Figure 1:**
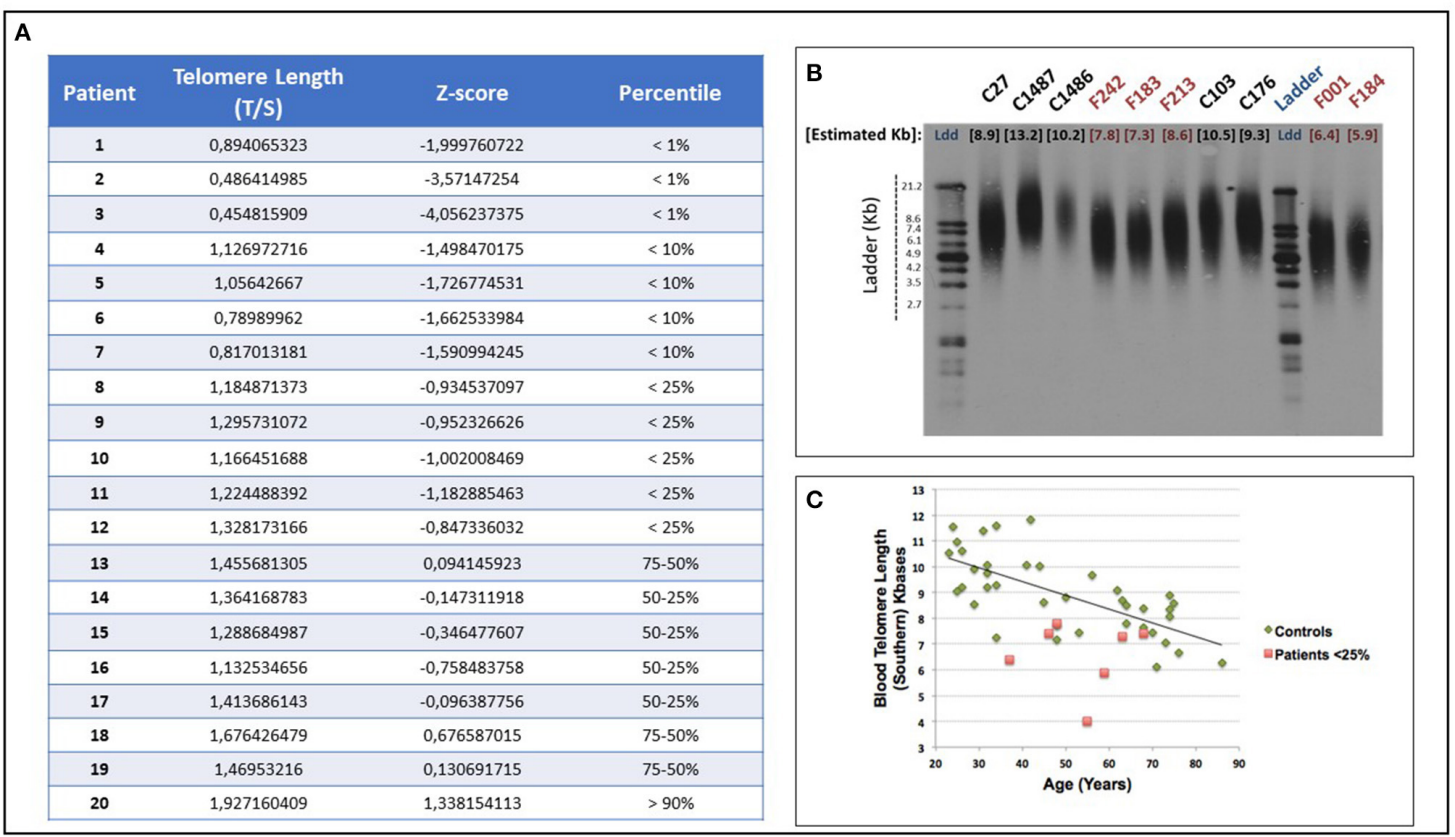
**(A)** Telomere length and qPCR essential primary data of all study patients: telomere length was measured, at diagnosis, from oral epithelium DNA, through quantitative Polymerase Chain Reaction (qPCR) and Z-score was obtained as described in Methods section. The qPCR determines the ratio of telomere (T) repeat copy number to single-copy (S) gene (*36B4*) copy number (T/S ratio), as compared with a reference DNA sample. The Z-score below the 25th percentile of a normal distribution was considered “telomere shortening.” **(B)** qPCR validation through TRF Southern Blot: telomere shortening was validated in each patient from blood DNA by telomere restriction fragment (TRF) Southern Blot analysis (TeloTAGGG Telomere Length Assay, Roche), which is considered the gold standard to determine telomere length. A representative southern blotting image is shown. The absolute telomere length of the telomeres in blood DNA samples from controls (C lanes) and patients (F lanes) was measured **(C)** Distribution of the absolute telomere length (kb) obtained by TRF Southern Blot in blood samples of controls and patients vs. age (years).

Telomere shortening was confirmed by telomere restriction fragment (TRF) assay (TeloTAGGG Telomere Length Assay, Roche) from peripheral blood DNA of each patient, as previously described (36, 37) (Figure 1). A positive correlation (*r* = 0.689) was observed between the two sets of telomere length measurements performed by either the qPCR on DNA extracted from buccal cells or by the Southern Blot of blood DNA, in accordance with a previous study published by Demanelis et al. (38).

The control population for telomere Z-score calculation consisted of 243 healthy subjects, with no respiratory disease, cancer, or any degenerative disorders such as diabetes, hematological, liver, or kidney disease. Other characteristics from the control population were described previously (19). Oral swab and peripheral blood sample were also processed in controls for DNA isolation. The same protocol described above was followed to process control DNA samples.

### Sequencing Analysis and Identification of Rare Coding Mutations in TRG

Patients with telomere shortening were subjected to whole-exome sequencing to obtain genetic variation at the telomere maintenance genes. Whole-exome sequences were obtained by the Instituto Tecnológico y de Energías Renovables (ITER). Briefly, DNA samples were processed with Nextera DNA Exome Kit with dual indexes following the manufacturer's recommendations (Illumina Inc., San Diego, CA). Library sizes were evaluated on a TapeStation 4200 (Agilent Technologies, Santa Clara, CA) and their concentration determined by the Qubit dsDNA HS Assay (Thermo Fisher, Waltham, MA). Samples were sequenced along with 1% of a PhiX control V3 to an average depth >50X (after removal of duplicate reads) on a HiSeq 4000 instrument (Illumina Inc.) with paired-end 75-base reads. Reads were preprocessed with bcl2fastq v2.18 and aligned (>99.70% of reads were mapped) to hg19 with BWA-MEM 0.7.15-r1140 (39). BAM files were processed with SAMtools v1.3 (40) and Picard v2.10.10, and small insertion/deletions (indels) and single nucleotide variants (SNVs) were identified with the HaplotypeCaller following the Best Practices workflow recommendations for germline variant calling in GATK (v3.8) (41). Detected variants were annotated for frequency in gnomAD, function and pathogenic potential with SnpEff v4.3 (42) and ANNOVAR v18.04.16 (43) based on data from different sources. We simplified the prioritization of pathogenicity potential by focusing only on the results in the previously-known TRG: *TERT* (chr5:1,253,262–1,295,184), *TERC* (chr3:169,482,308–169,482,848), *DCK1* (chrX:153,991,031–154,005,964), *PARN* (chr16:14,529,558–14,726,585), *RTEL1* (chr20:62,289,163–62,328,416), *TINF2* (chr14:24,708,849–24,711,880), and *TERF1* (chr8:73,921,097–73,960,357). Among the TRG set, we prioritized the variants that were likely to alter the function of the encoded proteins based on a CADD phred score >15 (standard threshold for deleteriousness) and rareness in the population (<0.5% in genomAD). On those patients that these criteria did not evidence a prioritized variant, we opted for prioritizing one variant applying more liberal filters but showing a predicted damage potential based on the effect (frameshift, stop gain/loss, splice site, codon deletion, non-synonymous change, etc.). Given the uncertainty in their pathogenic potential, we will refer to the prioritized variants as rare coding mutations thereafter.

### Statistical Analysis

Categorical variables were presented as the number of cases and percentages, while continuous variables were presented as the mean and SD or median and the interquartile range (IQR). In continuous variables, the group means were compared using the Student's *t*-test and the group medians were compared with the Mann-Whitney U-test. Fisher's exact test or a Pearson's *X*^2^-test were applied to assess the relationship between categorical variables. Time until death was assessed using the Kaplan–Meier estimator. Analyses were performed with R 3.4.0 (44). A *p*-value <0.005 was considered statistically significant.

## Results

### Patient Characteristics

From 274 fibrotic ILD patients that had been tested for telomere attrition at diagnosis, 20 cases underwent LT from June 2013 to October 2018. Telomere attrition was identified in 12 (60%) patients and rare coding mutations in TRG in 11 of them (55% of the cohort) (Supplementary Table 1). Patients underwent LT at a younger age if telomere dysfunction was present. Idiopathic pulmonary fibrosis and FPF were the most frequent diagnoses. The anti-fibrotic therapy was prescribed in 12 patients at diagnosis (Table 1).

**Table 1:** Patient characteristics.

	**All patients (*n* = 20)**	**With telomere shortening (*n* = 12)**	**Without telomere shortening (*n* = 8)**	***P*-value**
Age at lung transplant [years, *mean (SD)*]	56.6 (9.39)	51.8 (9.18)	63.8 (3.11)	0.001
Gender (*n*, %)				
- Males	15 (75.0)	9 (75.0)	6 (75.0)	1.000
- Females	5 (25.0)	3 (25.0)	2 (25.0)	
Smoking history at diagnosis (*n*, %):				
- Non-smoker	3 (15.0)	2 (16.7)	1 (12.5)	1.000
- Current smoker	3 (15.0)	2 (16.7)	1 (12.5)	
- Former smoker	14 (70.0)	8 (66.7)	6 (75.0)	
Diagnosis (*n*, %):				
- IPF	8 (40.0)	4 (33.3)	4 (50.0)	0.961
- FPF	8 (40.0)	5 (41.6)	3 (37.5)	
- HP	1 (5.0)	0 (0.0)	1 (12.5)	
- CPFE	1 (5.0)	1 (8.3)	0 (0.0)	
- DIP	1 (5.0)	1 (8.3)	0 (0.0)	
- CTD-ILD	1 (5.0)	1 (8.3)	0 (0.0)	
Pre-transplant treatment (*n*, %):				
- Pirfenidone	7 (35.0)	2 (16.6)	5 (62.5)	0.062
- Nintedanib	5 (25.0)	3 (25.0)	2 (25.0)	1.000
- Corticosteroids	4 (20.0)	2 (16.6)	2 (25.0)	1.000
- Corticosteroids + immunosuppressive drug	5 (25.0)	5 (41.6)	0 (0.0)	0.055
Telomere shortening (*n*, %):				
- *n*, below 1^st^ percentile	3 (25.0)	3 (25.0)	0 (0.0)	NA
- *n*, below 10^th^ percentile	4 (33.3)	4 (33.3)	0 (0.0)	
- *n*, below 25^th^ percentile	5 (41.7)	5 (41.7)	0 (0.0)	
Relative telomere length [*mean, (SD)*]	1.13 (0.33)	0.95 (0.29)	1.40 (0.17)	0.001
Z-score [*mean, (SD)*]	−1.15 (1.22)	−1.83 (1.04)	−0.006 (0.44)	0.001
Likely causal variants in telomere-maintenance genes (n =1 8) (*n*, %):	11 (55.0)	10 (83.3)	1 (12.5)	NA
- RTEL1	5 (45.5)	5 (50.0)	0 (0.0)	
- DKC1	2 (18.2)	2 (20.0)	0 (0.0)	
- PARN	1 (09.1)	0 (0.0)	1 (100)	
- TERT	3 (27.3)	3 (30.0)	0 (0.0)	

*IPF, idiopathic pulmonary fibrosis; FPF, familial pulmonary fibrosis; HP, hypersensivity pneumonitis; CPFE, combined pulmonary fibrosis and emphysema; DIP, desquamative interstitial pneumonia; CTD-ILD, connective tissue disease-associated interstitial lung disease; NA, not applicable*.

A similar pre-transplant functional status was reported for patients with or without telomere dysfunction (Table 2). Oxygen therapy was prescribed in all patients with telomere dysfunction (*n* = 12, 100%), with a median time of prescription of 12.5 months (range 11.0–16.2); and in 7 (87.5%) patients without telomere disorders with a median prescription time of 9.0 months (range 7.5–12.5) (*p* = 0.090). The pre-transplant health status questionnaires ATAQ and K-BILD were scored in the entire patient series. Patients with telomere dysfunction presented a worse pre-transplant health status compared to the patients without telomere disorders, with a median ATAQ score of 108 (8.27) and a median K-BILD score of 22.5 (16.0), (*p* = 0.032 and *p* = 0.036, respectively) (Table 2).

**Table 2:** Pre-transplant functional status.

	**All patients (*n* = 20)**	**With telomere shortening (*n* = 12)**	**Without telomere shortening (*n* = 8)**	***P*-value**
LAS [median, (Q1;Q3)]	40.3 (37.3;46.0)	43.1 (38.4;51.3)	39.1 (36.9;41.2)	0.283
Time from diagnosis to transplant [months, *mean (SD)*]	42.1 (32.1)	36.2 (36.7)	51.0 (23.3)	0.284
Time from diagnosis to transplant [months, *median* (Q1;Q3)]	33.1 (24.0;50.3)	26.5 (21.9;36.3)	51.2 (30.8;67.6)	0.076
Waiting list time [months, *median* (Q1;Q3)]	5.00 (1.75;8.00)	5.00 (2.75;7.25)	4.00 (1.00;9.25)	0.877
FVC [L, *mean (SD)*]	1.87 (0.44)	1.96 (0.39)	1.73 (0.50)	0.294
FVC [%, *mean (SD)*]	45.3 (11.1)	45.9 (13.8)	44.5 (5.92)	0.764
DLCO [%, *mean (SD)*]	22.8 (5.71)	21.2 (6.06)	24.7 (5.10)	0.255
Basal SatO_2_ <90% in WT6m [*n, mean (SD)*]	79.2 (5.76)	79.2 (5.14)	79.0 (6.97)	0.932
6-min walk test distance [*n, mean (SD)*]	292 (83.5)	274 (77.7)	319 (89.9)	0.267
Oxygen therapy (*n*, %)	19 (95)	12 (100)	7 (87.5)	0.400
Time with oxygen therapy before transplant [months, *mean (SD)*]	12.8 (5.55)	14.3 (6.02)	10.1 (3.63)	0.076
Time with oxygen therapy before transplant [months, *median (Q1;Q3)*]	12.0 (9.00;15.5)	12.5 (11.0;16.2)	9.00 (7.50;12.5)	0.090
ATAQ score [*mean (SD)*]	102 (13.3)	108 (8.27)	92.6 (14.4)	0.032
K-BILD score [*mean (SD)*]	28.4 (15.1)	22.5 (16.0)	36.7 (9.20)	0.036

*LAS, lung allocation score; FVC, forced vital capacity; DLCO, diffusion capacity for carbon monoxide; LT, lung transplant*.

Regarding the LAS score, the patients with telomere disorders had a median LAS of 43.1 (range 38.4–51.3) with no statistically significant difference from patients without telomere dysfunction (*p* = 0.283) (Table 2). Despite the median time from diagnosis to the LT was lower in those cases with telomere dysfunction (*p* = 0.076), the time on the waiting list was similar among the patients with and without telomere disorders (*p* = 0.877) (Table 2).

### Surgical Procedure and Immediate Post-surgery Requirements

Unilateral LT was performed in 14 (70.0%) cases. In both groups of patients, with and without telomere disorders, unilateral LT was the main choice.

Although no statistically significant differences were found during the surgical procedure, more cases with telomere dysfunction required extracorporeal circulation (ECC); among four patients, three of them presented telomere dysfunction. A longer time for ECC was registered for the telomere dysfunction group (median 150 min, range 122–244). The time of ischemia was also longer for patients with telomere disorders (median 6.88 h, range 5.25–7.69) (Table 3).

**Table 3:** Surgical technique and immediate post-operative requirements.

	**All patients (*n* = 20)**	**With telomere shortening (*n* = 12)**	**Without telomere shortening (*n* = 8)**	***P*-value**
Type of lung transplant (*n*, %):				1.000
- Unilateral	14 (70.0)	8 (66.7)	6 (75.0)	
- Bilateral	6 (30.0)	4 (33.3)	2 (25.0)	
Ischemia time [h, *mean (SD)*]	6.61 (2.82)	6.86 (2.55)	6.23 (3.33)	0.658
Ischemia time [h, *median (Q1;Q3)*]	6.42 (4.75;7.69)	6.88 (5.25;7.69)	5.42 (4.22;6.83)	0.354
Extracorporeal circulation need (*n*, %)	4 (20.0)	3 (25.0)	1 (12.5)	0.619
Extracorporeal circulation time [min, *median (Q1;Q3)*]	122 (88.0;197)	150 (122;244)	70.0 (70.0;70.0)	0.180
Stay in ICU [days, *median (Q1;Q3)*]	22.0 (7.50;36.5)	30.0 (7.75;40.8)	17.0 (7.00;22.5)	0.352
Post-operative complications in the first 24 h (*n*, %)	18 (90.0)	10 (83.3)	8 (100)	0.495
Difficult weaning (*n*, %) (*n* = 19)	10 (52.6)	6 (54.5)	4 (50.0)	1.000
Tracheostomy (*n*, %) (*n* = 19)	10 (52.6)	6 (54.5)	4 (50.0)	1.000

Regarding the first 24 h after LT, 15% of overall cases presented relevant complications, including hemodynamic instability, phrenic or diaphragmatic paresis, hypovolemic shock, thrombocytopenia, and primary graft dysfunction (Supplementary Table 2). No significant differences were observed by telomere dysfunction (*p* = 0.495) (Table 3). However, mild hematological complications during the first 24 h after LT were only reported in patients with telomere disorders (Supplementary Table 2).

Concerning the ICU admission period, the average stay was longer for patients with telomere dysfunction with a median of 30.0 days [(range 7.75–40.8 days, *p* = 0.352)] (Table 3). Time of respiratory support required, difficult weaning, and tracheostomy did not differ significantly due to the presence of telomere dysfunction (*p* = 1.000) (Table 3).

### Complications and Morbidity After Lung Transplant

We analyzed the complications reported and hospital admission requirements after LT. More than one complication per patient was reported regardless of telomere dysfunction (*p* = 1.000) (Table 4). During the first month, complications were present in 9 (81.8%) patients with telomere disorders, and in all patients (*n* = 8, 100%) without (*p* = 0.485) (Table 4). Renal failure was the main complication in the overall population (Supplementary Table 3). A higher rate of primary graft dysfunction and hypovolemic shock was more frequently reported for patients with telomere dysfunction; while phrenic or diaphragmatic paresis and hemodynamic instability were more present in patients without. The main hematological complication, thrombocytopenia, was only described in telomere dysfunction group, although pre-transplant thrombocytopenia was only present in one of those cases (Supplementary Table 3).

**Table 4:** Long-term complications after lung transplant.

	**All patients (*n* = 20)**	**With telomere shortening (*n* = 12)**	**Without telomere shortening (*n* = 8)**	***P*-value**
>1 complication after transplant (*n*, %)^*^ (*n* = 20)	18 (90.0)	11 (91.7)	7 (87.5)	1.000
Post-operative complications in the first 24 h (*n*, %)	18 (90.0)	10 (83.3)	8 (100)	0.495
Complications during the first month (*n*, %)	17 (89.5)	9 (81.9)	8 (100)	0.485
Complications after the first month (*n*, %) (*n* = 19)	18 (94.7)	11 (100)	7 (87.5)	0.421
Allograft dysfunction (*n*, %)	8 (42.1)	3 (27.3)	5 (62.5)	0.181
Nephrological complications (*n*, %)	7 (35.0)	4 (33.3)	3 (37.5)	1.000
Positive pre-transplant CMV serology (*n*, %)	18 (90.0)	11 (91.7)	7 (87.5)	1.000
Post-transplant CMV replication (*n*, %) (*n* = 19)	14 (73.7)	7 (63.6)	7 (87.5)	0.338
Hematological alterations due to CMV treatment (*n*, %) (*n* = 15)	4 (26.7)	3 (33.3)	1 (16.7)	0.604
CMV negativity after treatment (*n*, %) (*n* = 16)	9 (56.2)	4 (44.4)	5 (71.4)	0.358
Infectious complications (*n*, %)	12 (60.0)	6 (50.0)	6 (75.0)	0.373
Hematological complications (*n*, %)	6 (30.0)	4 (33.3)	2 (25.0)	1.000
Endocrine complications (*n*, %)	15 (75.0)	8 (66.7)	7 (87.5)	0.603
Hospital admissions after transplant [median (Q1;Q3)] (*n* = 19)	1.0 (0.00;3.00)	2.00 (1.00;3.00)	0.00 (0.00;1.25)	0.033

* *>1 complication at any time after lung transplant*.

After the first month of LT, all patients with telomere dysfunction (*n* = 11, 100%) and 7 (87.5%) patients without telomere disorders had complications (*p* = 0.421). As shown in Table 4, endocrine dysfunction and infections were predominant in both groups. Chronic lung allograft dysfunction (CLAD) and post-transplant CMV replication were higher in patients without telomere alterations [*n* = 5 (62.5%) and *n* = 7, (87.5%) respectively]. However, patients with telomere dysfunction showed a lower rate of CMV negativity after Valganciclovir (*n* = 4, 44.4%) with a higher rate of hematological complications due to CMV treatment (*n* = 3, 33.3%) (Table 4). Long-term complications are summarized in Supplementary Table 4. The type of complications according to telomere dysfunction and time after LT are summarized in Table 5.

**Table 5:** Post-transplant complications according to telomere dysfunction and time after lung transplantation.

**Type of post-transplant complications**	**First 24 h**		**During the first month**		**After the first month**	
	**Telomere shortening**	**No telomere shortening**	**Telomere shortening**	**No telomere shortening**	**Telomere shortening**	**No telomere shortening**
Endocrine (*n*, %)	0 (0.0)	0 (0.0)	0 (0.0)	0 (0.0)	8 (67)	7 (88)
Post-transplant CMV replication (*n*, %)	0 (0.0)	0 (0.0)	0 (0.0)	0 (0.0)	7 (58)	7 (88)
Infectious (*n*, %)	0 (0.0)	0 (0.0)	0 (0.0)	0 (0.0)	6 (50)	6 (75)
Allograft dysfunction (*n*, %)	0 (0.0)	0 (0.0)	0 (0.0)	0 (0.0)	3 (25)	5 (62)
Hemodynamic dysfunction (*n*, %)	4 (33)	3 (38)	3 (25)	3 (38)	0 (0.0)	0 (0.0)
Hematological (*n*, %)	4 (33)	0 (0.0)	2 (17)	0 (0.0)	4 (33)	2 (25)
Mechanical (*n*, %)	3 (25)	3 (38)	2 (17)	3 (38)	0 (0.0)	0 (0.0)
Primary graft dysfunction (*n*, %)	2 (17)	1 (12)	2 (17)	1 (12)	0 (0.0)	0 (0.0)
Donor lung infection (*n*, %)	0 (0.0)	1 (12)	0 (0.0)	1 (12)	0 (0.0)	0 (0.0)
Atrial fibrillation (*n*, %)	2 (17)	0 (0.0)	1 (8)	0 (0.0)	0 (0.0)	0 (0.0)
Renal (*n*, %)	1 (8)	1 (12)	0 (0.0)	0 (0.0)	4 (33)	3 (38)
Miopathy of critical patient (*n*, %)	1 (8)	0 (0.0)	1 (8)	0 (0.0)	0 (0.0)	0 (0.0)

Regarding hospital admissions after LT, patients with telomere dysfunction presented more hospitalizations (*p* = 0.033) and 2.9 times higher risk for readmission [Incidence Rate Ratio of 2.91, 95%CI = 1.11–7.59, (*p* = 0.029)] (Table 4). Infections were the main cause of readmission in both groups of patients (Table 6).

**Table 6:** Causes for hospital admission after lung transplant.

**Patient**	**Family aggregation**	**Percentile telomere length**	**Likely causal gene(s)**	**Hospital admissions after transplant**	**Infectious cause**	**Other causes**
**PATIENTS WITHOUT TELOMERE SHORTENING**						
1	Yes	75–50^th^	Not tested	1	Urinary sepsis	None
2	No	50–25^th^	Not tested	1	Lower right lobe pneumonia (*P. aeruginosa*)	None
3	No	50–25^th^	Not tested	1	Respiratory infection (Rhinovirus)	CLAD
**PATIENTS WITH TELOMERE SHORTENING**						
4	No	<25^th^	TERT	1	None	Cervical lymphadenectomy (M1 squamous cell carcinoma of unknown primary origin)
5	Yes	<25^th^	TERT	3	Respiratory infection (MRSA) Empyema (*S.aureus)*	Suspected unconfirmed CLAD
6	No	<25^th^	RTEL1	3	Acute gastroenteritis Elbow abscess Influenza A virus infection	None
7	Yes	<25^th^	DKC1	3	CMV viremia Lower right lobe pneumonia + non-complicated parapneumonic pleural effusion	Fever of unknown origin
8	No	<10^th^	RTEL1	1	None	CLAD
9	No	<10^th^	Not found	2	None	Pulmonary embolism Cervix squamous cell carcinoma stage IIIB
10	No	<10^th^	RTEL1	3	Respiratory infection (Respiratory syncytial virus) Bacteremia (*P.aeruginosa*) Influenza b virus infection	None
11	No	<1^th^	DKC1	6	Respiratory infection (x2) Perianal condylomatosis	Acute ILD exacerbation in native lung ILD progression in native lung (X2)
12	Yes	<1^th^	Not found	1	Cavitated pneumonia (*P.aeruginosa*) + bacteriemia (*P.aeruginosa*)	None

### Survival and Quality of Life After Lung Transplant

**Figure 2:**
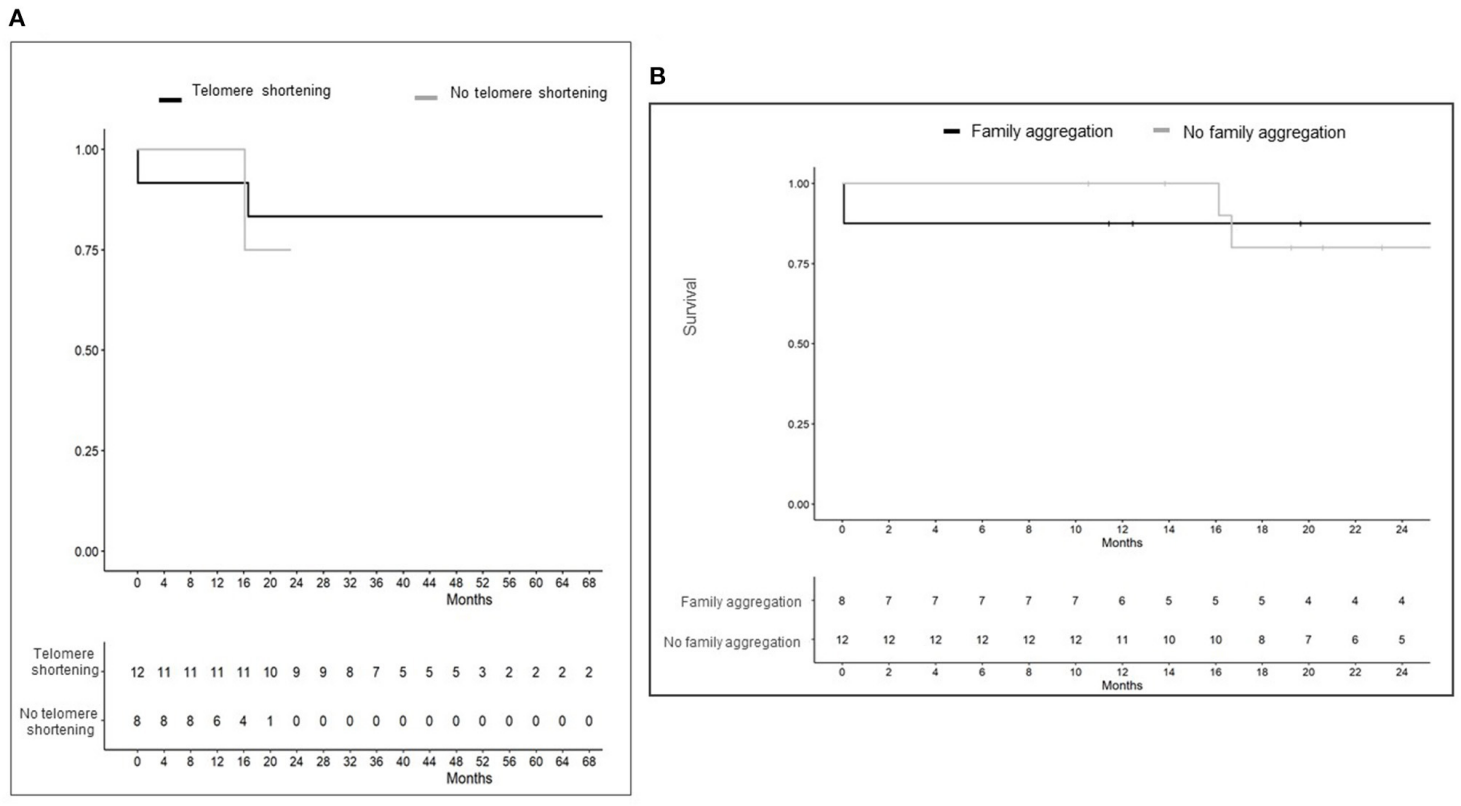
**(A)** Post-transplant survival according to presence or absence of telomere dysfunction. Patients with telomere dysfunction had a greater post-transplant follow up period due to an earlier requirement of lung transplant after pulmonary fibrosis diagnosis. As previously described in Table 2, a shorter time from diagnosis to transplantation was reported for patients with telomere dysfunction. **(B)** Survival after lung transplant according to family aggregation. A worse post-transplant survival was reported for family aggregation patients group during the first year. However, at 24 months, post-transplant survival was ≤ 50% regardless of family aggregation. Since genetic studies have not yet been implemented in routine clinical practice in most centers, further studies are needed to evaluate family aggregation as an outcome predictor regardless of the underlying genetic defect.

At the end of the study, 17 of 20 subjects (85%) survived after transplantation: 10/12 with telomere dysfunction (83.3%) and 7/8 (87.5%) without. Survival after LT is represented in Figure 2A. Patients with telomere shortening had a median post-transplant follow-up period of 39 months (range of 27–50 months) with two deaths reported. One subject died after bilateral LT due to multi-organic failure in the first 24 h; and the other patient died after 17 months of post-transplant follow-up due to stage IIIB cervical squamous-cell carcinoma. Both had a telomere length below the 10^th^ percentile and one of them carried a rare coding RTEL1 gene mutation (chr20:62317210). No TRG mutations were identified in the other patient. The median post-transplant follow-up period in patients without telomere dysfunction was 15 months (range of 12–19) with one death reported after 17 months of unilateral LT due to CLAD. The time from diagnosis to LT was shorter in those cases with telomere dysfunction. Therefore, we also analyzed survival from diagnosis to the end of the study period. No significant differences were found between both groups; 5 years (range 5–6 years) vs. 6 years (range 4–7 years), with and without telomere dysfunction, respectively.

**Figure 3:**
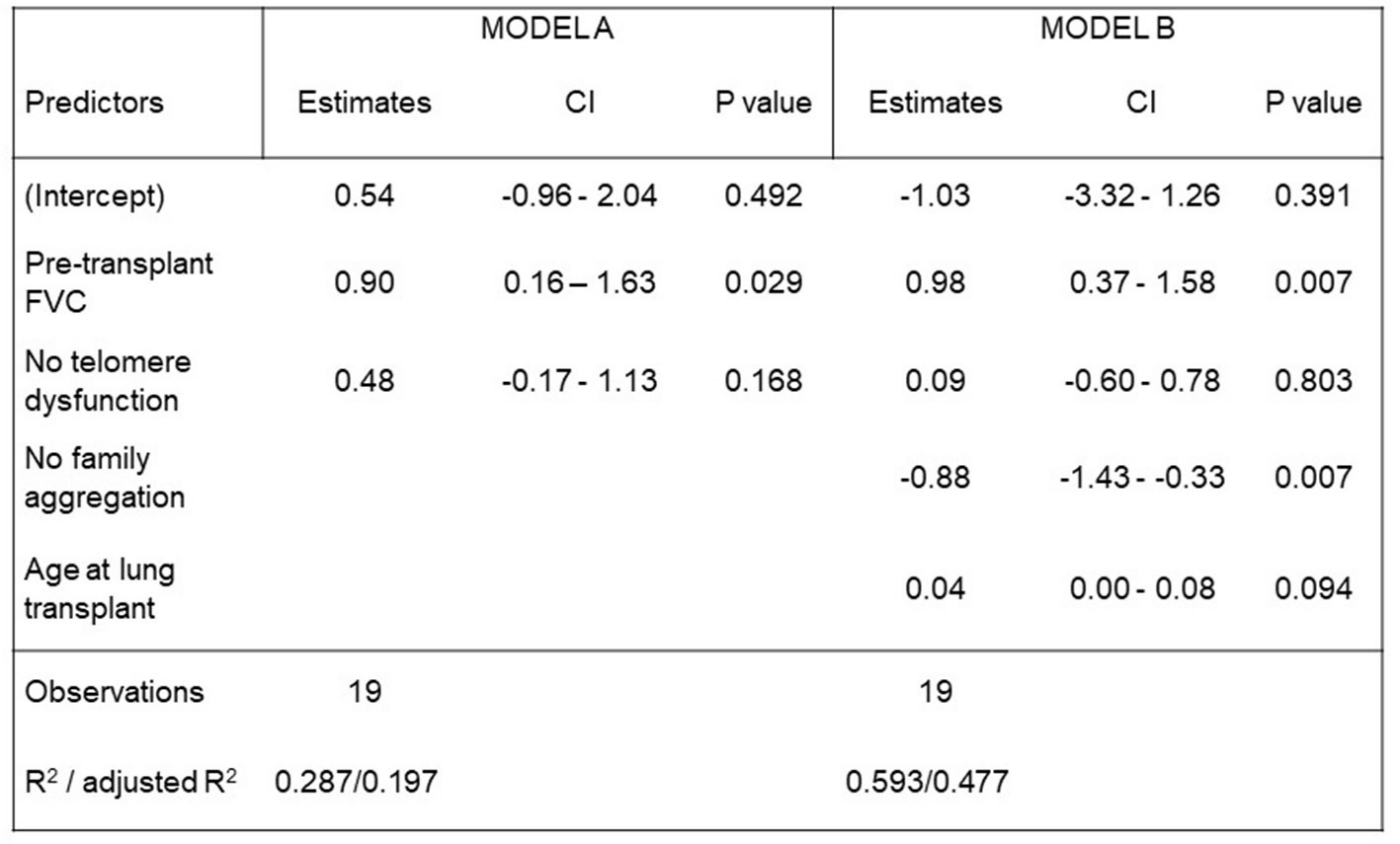
Post-transplant FVC (L) models. We analyzed the impact in post-transplant FVC of the following variables: the pre-transplant FVC, the age at the time of lung transplantation and the absence of telomere dysfunction and pulmonary fibrosis family aggregation. A statistically significant impact in post-transplant FVC was reported for pre-transplant FVC and the absence of family aggregation. The absence of telomere dysfunction did not statistically impact in post-transplant FVC.

Post-transplant quality of life assessment reported a significant improvement in respiratory functional and health status for all patients. The mean FVC increase after LT for the entire study population was 0.55 L (*p* = 0.0003) [95%CI: 0.21–0.89]. As shown in Figure 3, pre-transplant FVC-values (L) and the absence of family aggregation significantly influenced the post-transplant FVC-value (L) (*p* = 0.007, respectively). Regarding the health status, a significant improvement was reported overall through ATAQ-IPF and K-BILD scores, with *p* < 0.001 for both questionnaires. No differences in ATAQ-IPF and K-BILD post-transplant scores were found in telomere dysfunction patient group (Table 7). Likewise, no differences were found for declared daily life activity independence and active life after LT among patients with and without telomere disorders. Nine out of 10 (90%) patients with telomere dysfunction reported daily life activity independence, and 6 out of 10 (60%) had an active life (*p* = 1.000). In addition, oxygen therapy withdrawal was possible in 16 out of 17 (94.1%) patients of the overall population: 9 out of 10 (90.0%) with telomere dysfunction and 7 out of 7 (100%) without it (Table 7).

**Table 7:** Quality of life after lung transplant.

	**All patients (*n* = 20)**	**With telomere shortening (*n* = 12)**	**Without telomere shortening (*n* = 8)**	***P*-value**
Post-transplant FVC [ L, mean (*SD*)]	2.42 (0.77)	2.31 (0.51)	2.57 (1.05)	0.524
Post-transplant FVC [%, mean (*SD*)]	60.4 (17.6)	53.0 (12.7)	70.4 (19.0)	0.045
Post-transplant ATAQ score [mean (*SD*)]	58.2 (12.9)	60.6 (15.0)	54.7 (9.16)	0.333
Post-transplant K-BILD score [mean (*SD*)]	60.1 (9.14)	60.1 (9.66)	60.0 (9.10)	0.983
Oxygen withdrawal after transplantation (*n*, %)	16 (94.1)	9 (90.0)	7 (100)	1.000
Independence for daily life activities after transplantation (*n*, %)	15 (88.2)	9 (90.0)	6 (85.7)	1.000
Active life after transplantation (*n*, %)	11 (64.7)	6 (60.0)	5 (71.4)	1.000

*Independence for daily life activities: feeding, personal hygiene and dressing tasks without help. Active life: to be able to return to work and/or hobbies*.

### Lung Transplant in Familial Pulmonary Fibrosis

Family aggregation was reported in 8 (40.0%) patients and telomere shortening was present in 5 (62.5%) of them. Rare coding TRG mutations were identified in 4 out of 8 (50.0%) patients. A similar pre-transplant functional status was reported according to family aggregation without differences in the time from diagnosis to the LT (Supplementary data Table 5). FPF cases presented a non-significant higher rate of difficult weaning and a greater need for tracheostomy. No differences were found in the ratio of the other complications during the first 24 h and the first month. Long-term complications were reported in all patients. Those cases without family aggregation presented a trend of higher rate for mechanical, nephrological, hematological, endocrine, and infectious complications (Supplementary Table 5). Survival after transplantation according to family aggregation is represented in Figure 2B.

## Discussion

Previous studies have analyzed LT morbidity and mortality in patients with telomere abnormalities with controversial results (19–25). Our study reveals for the first time a long-term positive LT outcome in overall fibrotic ILD population regardless of telomere dysfunction. Furthermore, we report the order in appearance and the frequency of the subsequent complications according to elapsed time from transplantation and presence of telomere dysfunction. Therefore, while a higher rate of LT complications has previously been reported in patients with telomeropathy (19–25), our study highlights the long-term benefits of LT despite telomere dysfunction by optimizing the knowledge and the management of transplant morbidity.

Our main finding was the benefit of LT in survival of fibrotic ILD regardless of telomere dysfunction. We report a good LT outcome in the overall study population with a survival rate >80% and a similar post-transplant follow-up. Previous studies have also evaluated the impact of transplantation for pulmonary fibrosis in ILD patients carrying telomere disorders. Swaminathan et al. reported a significantly higher risk of death for patients carrying variants in *TERT, RTEL1*, or *PARN* (25); while Newton et al. showed the association between telomere length below the 10^th^ percentile and worse post-transplant survival (23). Although this poor outcome was related to systemic complications of telomere dysfunction, Borie et al. reported no differences in survival between controls and telomerase mutation carriers with higher risk for hematological complications (21). Similarly, Silhan et al. also described a reasonable post-transplant survival despite a higher rate of post-transplant complications (20). In addition, LT feasibility was also assessed for other telomeropathies like dyskeratosis congenital (45) and in the case of multiple solid organ transplantation (46) with a favorable long-term post-transplant outcome.

The controversial results reported so far are probably due to a wide dispersion of methods and lack of control group in most studies (19–25). Moreover, a broad variety of transplant centers were involved in the previous studies with great heterogeneity of national organ transplant programs and center-specific pre-surgical, surgical, and post-surgical procedures. Very few randomized studies have been conducted in LT for the general population leaving some topics with a lack of evidence and outcomes far from optimal for many patients (47). Hence, making decisions in LT is sometimes highly controversial and requires an evidence-experience balance. Considering these arguments, we prospectively assessed LT outcomes according to telomere dysfunction as the only differential biological marker among recipients in our single-transplantation center cohort. No differences were found in pre-transplant functional status for the overall study population despite the younger mean age of the telomere dysfunction group of patients, the fibrotic ILD and/or the pre-transplant treatment prescription.

An important issue when considering survival is to investigate the quality of patient survival. Although morbidity and mortality have previously been assessed in fibrotic ILD with telomeropathy (20–25), knowledge is lacking about outcomes in terms of quality of life. Our study describes for the first time the post-transplant benefits in quality of life despite telomere disorders and their complications. The enhancement of post- transplant quality of life was reported for the overall study population through the improvement of ATAQ and K-BILD scores and the feasibility for independent and active life with oxygen withdrawal. Hence, our study shows quality of life gains for transplanted ILD patients regardless of telomere dysfunction.

However, this favorable effect needs to be balanced with the higher rate of post-transplant morbidity reported for telomeropathies. Therefore, our research thoroughly investigates the morbidity profiles according to elapsed time from transplantation. The post-transplant appearance and predominance of complications could be chronological and change according to successive clinical requirements after transplantation and patient susceptibility. Telomere dysfunction is associated with immunodeficiency (28) and poor outcomes of immunosuppressive drugs (26, 27). Thereby, it could explain our higher rate of hospital admissions after transplantation for the telomere dysfunction group due to a wide variety of infections. The post-transplant antibiotic prophylaxis in these cases is a hypothesis that still needs to be evaluated. The CMV post-transplant prophylaxis performed could explain a similar rate of CMV relapsing viremia in those cases with or without telomere dysfunction, deviating from previous reports (24). Although a similar effect, CMV prophylaxis toxicity was mainly reported in telomere dysfunction cases as thrombocytopenia or leukopenia. Hematological complications are highly associated with telomeropathy (20, 21) and may complicate further due to ECC in the early post-transplant period and long-term drug toxicities.

The main limitations of the present study are the small sample size of our series and the lack of a validation cohort. However, LT experience and protocols may differ among centers, which represents a major difficulty for homogeneously increasing the number of cases or finding a proper validating cohort with patients screened for telomere length at diagnosis.

Hence, increasing the knowledge about when and why each complication appears would set specific prevention and management strategies in order to tip the balance in favor of survival benefits. Pre-transplant work-up considerations should include telomere disorders in order to establish variations in care (48). However, wider evidence is clearly needed to identify interventions that work best for this subset of ILD patients.

In conclusion, our results support the survival gains of lung transplant regardless of telomere disorders.

## Data Availability Statement

The original contributions presented in the study are publicly available. This data can be found in the European Variation Archive (EVA), accession numbers PRJEB46414 and ERZ2875159.

## Ethics Statement

The studies involving human participants were reviewed and approved by Ethics Committee of Hospital Universitari de Bellvitge. The patients/participants provided their written informed consent to participate in this study.

## Author Contributions

The participation level of each author is the following: LP-C and MM-M had full access to all of the data in the study, and take responsibility for the integrity of the data and the accuracy of the data analysis, including and especially any adverse effects, and contributed to statistical analysis and interpretation. LP-C served as principal author. MM-M served as corresponding author. LP-C, EA-S, CB, RP, AM-W, CF, AR, and MM-M contributed to the study concept and design. LP-C, CB, VV-Z, MG-M, AR, and MM-M contributed to identification and inclusion of patients and clinical data collection. LP-C, EA-S, AM-W, RG-M, JL-S, VV-Z, MG-M, JD, CF, RP, AR, and MM-M contributed to patient's sample collection and processing. LP-C, EA-S, AM-W, RG-M, JL-S, CF, RP, and MM-M contributed to analysis and interpretation of data for the work. LP-C, EA-S, CB, RG-M, JL-S, AM-W, CF, RP, AR, and MM-M contributed to the writing of the manuscript. All authors contributed to review critically of the draft and approved the final manuscript.

## Conflict of Interest

The authors declare that the research was conducted in the absence of any commercial or financial relationships that could be construed as a potential conflict of interest.

## Publisher's Note

All claims expressed in this article are solely those of the authors and do not necessarily represent those of their affiliated organizations, or those of the publisher, the editors and the reviewers. Any product that may be evaluated in this article, or claim that may be made by its manufacturer, is not guaranteed or endorsed by the publisher.

## References

[1] Cottin V Wollin L Fischer A Quaresma M Stowasser S Harari S. Fibrosing interstitial lung diseases: knowns and unknowns. Eur Respir Rev. (2019) 28:180100. 10.1183/16000617.0100-2018 30814139 PMC9489101

[2] Raghu G Collard HR Egan JJ Martinez FJ Behr J Brown KK. An official ATS/ERS/JRS/ALAT statement: idiopathic pulmonary fibrosis: evidence-based guidelines for diagnosis and management. Am J Respir Crit Care Med. (2011) 183:788–824. 10.1164/rccm.2009-040GL 21471066 PMC5450933

[3] Chambers DC Cherikh WS Goldfarb SB Hayes D Jr Kucheryavaya AY Toll AE. The International Thoracic Organ Transplant Registry of the International Society for Heart and Lung Transplantation: thirty-fifth adult lung and heart-lung transplant report-2018; focus theme: multiorgan transplantation. J Heart Lung Transplant. (2018) 37:1169–83. 10.1016/j.healun.2018.07.020 30293613

[4] Armanios M. Syndromes of telomere shortening. Annu Rev Genomics Hum Genet. (2009) 10:45–61. 10.1146/annurev-genom-082908-150046 19405848 PMC2818564

[5] Armanios M Telomerase and idiopathic pulmonary fibrosis. Mutat Res (2012) 730:52–8. 10.1016/j.mrfmmm.2011.10.013 22079513 PMC3292861

[6] Armanios M Blackburn E. The telomere syndromes. Nat Rev Genet. (2012) 13:693–704. 10.1038/nrg3246 22965356 PMC3548426

[7] Townsley DM Dumitriu B Young NS. Bone marrow failure and the telomeropathies. Blood. (2014) 124:2775–83. 10.1182/blood-2014-05-526285 25237198 PMC4215309

[8] Armanios M. Telomeres and age-related disease: how telomere biology informs clinical paradigms. J Clin Invest. (2013) 123:996–1002. 10.1172/JCI66370 23454763 PMC3673231

[9] Alder JK Cogan JD Brown AF Anderson CJ Lawson WE Lansdorp PM. Ancestral mutation in telomerase causes defects in repeat addition processivity and manifests in familial pulmonary fibrosis. PLoS Genet. (2011) 7:e1001352. 10.1371/journal.pgen.1001352 21483807 PMC3069110

[10] Tsakiri KD Cronkhite JT Kuan PJ Xing C Raghu G Weissler JC. Adult-onset pulmonary fibrosis caused by mutations in telomerase. Proc Natal Acad Sci USA. (2007) 104:7552–7. 10.1073/pnas.0701009104 17460043 PMC1855917

[11] Hao LY Armanios M Strong MA Karim B Feldser DM Huso D. Short telomeres, even in the presence of Telomerase, limit tissue renewal capacity. Cell. (2005) 123:1121–31. 10.1016/j.cell.2005.11.020 16360040

[12] Armanios M Alder JK Parry EM Karim B Strong MA Greider CW. Short telomeres are sufficient to cause the degenerative defects associated with aging. Am J Hum Genet. (2009) 85:823–32. 10.1016/j.ajhg.2009.10.028 19944403 PMC2790562

[13] Selman M Pardo A. Revealing the pathogenic and aging-related mechanisms of the enigmatic idiopathic pulmonary fibrosis. Am J Respir Crit Care Med. (2014) 189:1161–72. 10.1164/rccm.201312-2221PP 24641682

[14] Von Zglinicki T Martin-Ruiz CM. Telomeres as biomarkers for ageing and age-related diseases. Curr Mol Med. (2005) 5:197–203. 10.2174/1566524053586545 15974873

[15] Snetselaar R van Moorsel CHM Kazemier KM van der Vis JJ Zanen P van Oosterhout MFM. Telomere length in interstitial lung diseases. Chest. (2015) 148:1011–8. 10.1378/chest.14-3078 25973743

[16] Borie R Kannengiesser C Nathan N Tabèze L Pradère P Crestani B. Familial pulmonary fibrosis. Rev Mal Respir. (2015) 32:413–34. 10.1016/j.rmr.2014.07.017 25596800

[17] Newton CA Batra K Torrealba J Kozlitina J Glazer CS Aravena C. Telomere-related lung fibrosis is diagnostically heterogeneous but uniformly progressive. Eur Respir J. (2016) 48:1710–20. 10.1183/13993003.00308-2016 27540018 PMC5433348

[18] Molina-Molina M Borie R. Clinical implications of telomere dysfunction in lung fibrosis. Curr Opin Pulm Med. (2018) 24:440–4. 10.1097/MCP.0000000000000506 30067250

[19] Planas-Cerezales L Arias-Salgado EG Buendia-Roldán I Montes-Worboys A López CE Vicens-Zygmunt V. Predictive factors and prognostic effect of telomere shortening in pulmonary fibrosis. Respirology. (2019) 24:146–53. 10.1111/resp.13423 30320420

[20] Silhan LL Shah PD Chambers DC Snyder LD Riise GC Wagner CL. Lung transplantation in telomerase mutation carriers with pulmonary fibrosis. Eur Respir J. (2014) 44:178–87. 10.1183/09031936.00060014 24833766 PMC4076528

[21] Borie R Kannengiesser C Hirschi S Le Pavec J Mal H Bergot E. Groupe d'Etudes et de Recherche sur les Maladies “Orphelines” Pulmonaires (GERM“O”P). Severe hematologic complications after lung transplantation in patients with telomerase complex mutations. J Heart Lung Transplant. (2015) 34:538–46. 10.1016/j.healun.2014.11.010 25612863

[22] Tokman S Singer JP Devine MS Westall GP Aubert JD Tamm M. Clinical outcomes of lung transplant recipients with telomerase mutations. J Heart Lung Transplant. (2015) 34:1318–24. 10.1016/j.healun.2015.05.002 26169663 PMC5382798

[23] Newton CA Kozlitina J Lines JR Kaza V Torres F Garcia CK. Telomere length in patients with pulmonary fibrosis associated with chronic lung allograft dysfunction and post-lung transplantation survival. J Heart Lung Transplant. (2017) 36:845–53. 10.1016/j.healun.2017.02.005 28262440 PMC5515686

[24] Popescu I Mannem H Winters SA Hoji A Silveira F McNally E. Impaired cytomegalovirus immunity in idiopathic pulmonary fibrosis lung transplant recipients with short telomeres. Am J Respir Crit Care Med. (2019) 199:362–76. 10.1164/rccm.201805-0825OC 30088779 PMC6363970

[25] Swaminathan AC Neely ML Frankel CW Kelly FL Petrovski S Durheim MT. Lung transplant outcomes in patients with pulmonary fibrosis with telomere-related gene variants. Chest. (2019) 156:477–85. 10.1016/j.chest.2019.03.030 30978332

[26] Newton CA Zhang D Oldham JM Kozlitina J Ma SF Martinez FJ. Telomere length and use of immunosuppressive medications in idiopathic pulmonary fibrosis. Am J Respir Crit Care Med. (2019) 200:336–47. 10.1164/rccm.201809-1646OC 30566847 PMC6680304

[27] Molina-Molina M. Telomere shortening behind the harm of immunosuppressive therapy in idiopathic pulmonary fibrosis. Am J Respir Crit Care Med. (2019) 200:274–5. 10.1164/rccm.201812-2330ED 30624965 PMC6680301

[28] Wagner CL Hanumanthu VS Talbot CC Jr Abraham RS Hamm D Gable DL. Short telomere syndromes cause a primary T cell immunodeficiency. J Clin Invest. (2018) 128:5222–34. 10.1172/JCI120216 30179220 PMC6264634

[29] American Thoracic Society European Respiratory Society. American Thoracic Society/European Respiratory Society international multidisciplinary consensus classification of the idiopathic interstitial pneumonias. Am J Respir Crit Care Med. (2002) 165:277–304. 10.1164/ajrccm.165.2.ats01 11790668

[30] Travis WD Costabel U Hansell DM King TE Jr Lynch DA Nicholson AG. An official American Thoracic Society/European Respiratory Society statement: update of the international multidisciplinary classification of the idiopathic interstitial pneumonias. Am J Respir Crit Care Med. (2013) 188:733–48. 10.1164/rccm.201308-1483ST 24032382 PMC5803655

[31] Raghu G Remy-Jardin M Myers JL Richeldi L Ryerson CJ Lederer DJ. Diagnosis of idiopathic pulmonary fibrosis. An official ATS/ERS/JRS/ALAT clinical practice guideline. Am J Respir Crit Care Med. (2018) 198:e44–68. 10.1164/rccm.201807-1255ST 30168753

[32] Orens JB Estenne M Arcasoy S Conte JV Corris P Egan JJ. International guidelines for the selection oflungtransplantcandidates: 2006 update—a consensus report from the Pulmonary Scientific Council of the International Society for Heart and Lung Transplantation. J Heart Lung Transplant. (2006) 25:745–55. 10.1016/j.healun.2006.03.011 16818116

[33] Weill D Benden C Corris PA Dark JH Davis RD Keshavjee S. A consensus document for the selection of lung transplant candidates: 2014. An update from the Pulmonary Transplantation Council of the International Society for Heart and Lung Transplantation. J Heart Lung Transplant. (2015) 34:1–15. 10.1016/j.healun.2014.06.014 25085497

[34] McMichael GL Gibson CS O'Callaghan ME Goldwater PN Dekker GA Haan EA. DNA from buccal swabs suitable for high-throughput SNP multiplex analysis. J Biomol Tech. (2009) 20:232–5. 19949693 PMC2777348

[35] Cawthon RM. Telomere measurement by quantitative PCR. Nucleic Acids Res. (2002) 30:e47. 10.1093/nar/30.10.e47 12000852 PMC115301

[36] Arias-Salgado EG Galvez E Planas-Cerezales L Pintado-Berninches L Vallespin E Martinez P. Genetic analyses of aplastic anemia and idiopathic pulmonary fibrosis patients with short telomeres, possible implication of DNA-repair genes. Orphanet J Rare Dis. (2019) 14:82. 10.1186/s13023-019-1046-0 30995915 PMC6471801

[37] Carrillo J Martínez P Solera J Moratilla C González A Manguán-García C. High resolution melting analysis for the identification of novel mutations in DKC1 and TERT genes in patients with dyskeratosis congenita Blood Cells Mol Dis. (2012) 49:140–6. 10.1016/j.bcmd.2012.05.008 22664374

[38] Demanelis K Jasmine F Chen LS Chernoff M Tong L Delgado D. Determinants of telomere length across human tissues. Science. (2020) 369:eaaz6876. 10.1126/science.aaz6876 32913074 PMC8108546

[39] Li H Durbin R. Fast and accurate long-read alignment with Burrows-Wheeler transform. Bioinformatics. (2010) 26:589–95. 10.1093/bioinformatics/btp698 20080505 PMC2828108

[40] Li H Handsaker B Wysoker A Fennell T Ruan J Homer N. 1000 Genome Project Data Processing Subgroup. The Sequence Alignment/Map format and SAMtools. Bioinformatics. (2009) 25:2078–9. 10.1093/bioinformatics/btp352 19505943 PMC2723002

[41] DePristo MA Banks E Poplin R Garimella KV Maguire JR Hartl C. A framework for variation discovery and genotyping using next-generation DNA sequencing data. Nat Genet. (2011) 43:491–8. 10.1038/ng.806 21478889 PMC3083463

[42] Cingolani P Platts A Wang le L Coon M Nguyen T Wang L. A program for annotating and predicting the effects of single nucleotide polymorphisms, SnpEff: SNPs in the genome of *Drosophila melanogaster* strain w1118; iso-2; iso-3. Fly (Austin). (2012) 6:80–92. 10.4161/fly.19695 22728672 PMC3679285

[43] Yang H Wang K. Genomic variant annotation and prioritization with ANNOVAR and wANNOVAR. Nat Protoc. (2015) 10:1556–66. 10.1038/nprot.2015.105 26379229 PMC4718734

[44] R Core Team. R: A Language and Environment for Statistical Computing. Vienna: R Found Stat Comput. Available online at: http//wwwR-Project.org/ (accessed February 10, 2015).

[45] Giri N Lee R Faro A Huddleston CB White FV Alter BP. Lung transplantation for pulmonary fibrosis in dyskeratosis congenita: case report and systematic literature review. BMC Blood Disord. (2011) 11:3. 10.1186/1471-2326-11-3 21676225 PMC3141321

[46] Lebeer M Wuyts WA Cassiman D Laleman W Nevens F Pirenne J. Multiple solid organ transplantation in telomeropathy: case series and literature review. Transplantation. (2018) 102:1747–55. 10.1097/TP.0000000000002198 29596117

[47] Abelson D Glanville AR. Controversies and emerging topics in lung transplantation. Breathe (Sheff). (2018) 14:278–87. 10.1183/20734735.027018 30519294 PMC6269170

[48] Courtwright AM Lamattina AM Takahashi M Trindade AJ Hunninghake GM Rosas IO. Shorter telomere length following lung transplantation is associated with clinically significant leukopenia and decreased chronic lung allograft dysfunction-free survival. ERJ Open Res. (2020) 6:00003-2020. 10.1183/23120541.00003-2020 32577419 PMC7293991

