# Supplementary material for: Lung Transplant Improves Survival and Quality of Life Regardless of Telomere Dysfunction
Source: Front Med (Lausanne). 2021 Jul 30;8:695919. doi: 10.3389/fmed.2021.695919 (PMC8362799; doi:10.3389/fmed.2021.695919)
Supplement: Supplementary file 1 [file Table_1.DOCX]

**SUPPLEMENTARY DATA:**

**Table 1. Rare coding mutations in telomere-maintenance genes identified in the study patients**

| Patient | Telomere shortening (percentile) | Chr. | Position^a^ | Ref.^b^ | Alt.^b^ | Gene | rs^c^ | CADD score | Frequency in gnomAD | Predicted effect |
| --- | --- | --- | --- | --- | --- | --- | --- | --- | --- | --- |
| 1 | < 1% | X | 154005088 | AAG | - | DKC1 | rs782576893 | 16.30 | 0.0038 | Codon deletion |
| 2 | < 1% | - | - | - | - | - | - | - | - | - |
| 3 | < 1% | 20 | 62324564 | C | T | RTEL1 | rs398123017 | 37.00 | 3.208x10^-5^ | Stop gain |
| 4 | < 10% | - | - | - | - | - | - | - | - | - |
| 5 | < 10% | 20 | 62317210 | G | A | RTEL1 | rs757365506 | 14.44 | 0.0002 | Non-synonymous coding |
| 6 | < 10% | 20 | 62325833 | C | A | RTEL1 | rs115610405 | 16.95 | 0.0163 | Non-synonymous coding |
| 7 | < 10% | 20 | 62325833 | C | A | RTEL1 | rs115610405 | 16.95 | 0.0163 | Non-synonymous coding |
| 8 | < 25% | 5 | 1279520 | CGC | - | TERT | - | - | - | Codon deletion |
| 9 | < 25% | 20 | 63693247 | C | T | RTEL1 | rs369419645 | 19.35 | 0.00004 | Stop gain |
| 10 | < 25% | 5 | 1294166 | C | T | TERT | rs61748181 | 8.24 | 0.0231 | Non-synonymous coding |
| 11 | < 25% | X | 154005088 | AAG | - | DKC1 | rs782576893 | 16.30 | 0.0038 | Codon deletion |
| 12 | < 25% | 5 | 1254611 | A | G | TERT | - | - | - | Non-synonymous coding |
| 13 | 75-50% | 16 | 14540938 | CTG | - | PARN | rs1233589581 | - | 2.048x10^-5^ | Splice site acceptor |

^a^According to human reference genome hg19; ^b^Allelic variants found in the reference (Ref.) and in the patient (Alt.); ^c^Reference sequence identifier, if available.

**Table 2. Postoperative complications during the first 24 hours after lung transplant**

|  | All patients  (n=20) (%) | With telomere shortening (n=12) (%) | Without telomere shortening (n=8) (%) |
| --- | --- | --- | --- |
| Mechanical complications:  -Phrenic or diaphragmatic paresis  -Atrial tear  -Suture failure  -Hemothorax | 3 (15.0)  1 (5.0)  1 (5.0)  1 (5.0) | 1 (8.3)  0 (0.0)  1 (8.3)  1 (8.3) | 2 (25.0)  1 (12.5)  0 (0.0)  0 (0.0) |
| Hemodynamic complications:  -Hemodinamic instability  -Hypovolemic shock  -Distributive shock | 3 (15.0)  3 (15.0)  1 (5.0) | 1 (8.3)  3 (25.0)  0 (0.0) | 2 (25.0)  0 (0.0)  1 (12.5) |
| Hematological complications:  -thrombocytopenia  -Coagulation alterations | 3 (15.0)  1 (5.0) | 3 (25.0)  1 (8.3) | 0 (0.0)  0 (0.0) |
| Primary graft dysfunction | 3 (15.0) | 2 (16.7) | 1 (12.5) |
| Renal failure | 2 (10.0) | 1 (8.3) | 1 (12.5) |
| Atrial fibrillation | 2 (10.0) | 2 (16.7) | 0 (0.0) |
| Donor lung infection | 1 (5.0) | 0 (0.0) | 1 (12.5) |
| Myopathy of the critical patient | 1 (5.0) | 1 (8.3) | 0 (0.0) |

**Table 3. Postoperative complications during the first month after lung transplantation**

|  | All patients  (n=20) (%) | With telomere shortening (n=12) (%) | Without telomere shortening (n=8) (%) |
| --- | --- | --- | --- |
| Mechanical complications:  -Phrenic or diaphragmatic paresis  -Atrial tear  -Suture failure | 3 (15.0)  1 (5.0)  1 (5.0) | 1 (8.3)  0 (0.0)  1 (8.3) | 2 (25.0)  1 (12.5)  0 (0.0) |
| Hemodynamic complications:  -Hemodinamic instability  -Hypovolemic shock  -Distributive shock | 3 (15.0)  2 (10.0)  1 (5.0) | 1 (8.3)  2 (16.7)  0 (0.0) | 2 (25.0)  0 (0.0)  1 (12.5) |
| Hematological complications:  -Plaquetopenia | 2 (10.0) | 2 (16.7) | 0 (0.0) |
| Primary graft dysfunction | 3 (15.0) | 2 (16.7) | 1 (12.5) |
| Renal failure | 20 (100.0) | 12 (100.0) | 8 (100.0) |
| Atrial fibrillation | 1 (5.0) | 1 (8.3) | 0 (0.0) |
| Donor lung infection | 1 (5.0) | 0 (0.0) | 1 (12.5) |
| Myopathy of the critical patient | 1 (5.0) | 1 (8.3) | 0 (0.0) |

**Table 4. Type of long-term complications after lung transplant**

|  | All patients  (n=19) (%) | With telomere shortening (n=12) (%) | Without telomere shortening (n=8) (%) | P value |
| --- | --- | --- | --- | --- |
| Allograft dysfunction  Type:  -Acute A1-A2  -Acute A2-A3  -Acute A3-A4  -Chronic | 8 (42.1)  1 (12.5)  4 (50.0)  2 (25.0)  1 (12.5) | 3 (27.3)  1 (33.3)  1 (33.3)  0 (0.0)  1 (33.3) | 5 (62.5)    0 (0.0)  3 (60.0)  2 (40.0)  0 (0.0) | 0.181  0.286 |
| Renal complications  Type:  -Renal failure  -Ureteral lithiasis + hydronephrosis | 7 (35.0)  6 (85.7)  1 (14.3) | 4 (33.3)  4 (100)  0 (0.0) | 3 (37.5)    2 (66.7)  1 (33.3) | 1.000  0.429 |
| Hematological complications  Type:  -Plaquetopenia  -Leukopenia  -Anemia | 6 (30.0)  4 (66.7)  1 (16.7)  1 (16.7) | 4 (33.3)  3 (75.0)  0 (0.0)  1 (25.0) | 2 (25.0)  1 (50.0)  1 (50.0)  0 (0.0) | 1.000  0.600 |
| Endocrine complications  Type:  -Diabetes due to corticoids  -Dyslipidemia  -Cushing Syndrome | 15 (75.0)  13 (86.7)  1 (6.67)  1 (6.67) | 8 (66.7)  7 (87.5)  1 (12.5)  0 (0.0) | 7 (87.5)  6 (85.7)  0 (0.0)  1 (14.3) | 0.603  1.000 |
| Infectious complications  Type:  -Surgical wound infection by *S.aureus*  -*P. aeruginosa* in BAL  -Catheter-related bacteriemia due to  *S. epidermidis* + urinary tract infection  due to *M. morganii*  - Catheter-related bacteriemia due to  *S. epidermidis*  - Catheter-related bacteriemia due to  *S. hominis*  -Urinary tract infection  -Endocarditis  -Septic thrombophlebitis  -Septic shock  -Respiratory infection | 12 (60.0)  1 (8.3)  1 (8.3)  1 (8.3)  1 (8.3)  1 (8.3)  1 (8.3)  1 (8.3)  1 (8.3)  2 (16.7)  2 (16.7) | 6 (50.0)  1 (16.7)  0 (0.0)  1 (16.7)  1 (16.7)  0 (0.0)  1 (16.7)  0 (0.0)  0 (0.0)  0 (0.0)  2 (33.3) | 6 (75.0)  0 (0.0)  1 (16.7)  0 (0.0)  0 (0.0)  1 (16.7)  0 (0.0)  1 (16.7)  1 (16.7)  2 (33.3)  0 (0.0) | 0.373  0.212 |
| CMV infection  - Positive pre-transplant CMV serology  - Post-transplant CMV replication  - Hematological alterations due to CMV  treatment  - CMV negativity after treatment | 18 (90.0)  14 (73.7)  4 (26.7)  9 (56.2) | 11 (91.7)  7 (63.6)  3 (33.3)  4 (44.4) | 7 (87.5)  7 (87.5)  1 (16.7)  5 (71.4) | 0.338  0.604  0.358 |

BAL = broncho-alveolar lavage

**Table 5. Post-transplant complications according to family aggregation**

|  | All patients  (n=20) | Patients with family aggregation (n=8) | Patients without family aggregation (n=12) |
| --- | --- | --- | --- |
| Presence of telomere shortening [n, %] | 12 (60.0) | 5 (62.5) | 7 (58.3) |
| Pre-transplant FVC  [liters, *mean (SD)*] | 1.87 (0.44) | 1.79 (0.31) | 1.92 (0.52) |
| Pre-transplant FVC  [%, *mean (SD)*] | 45.3 (11.1) | 39.7 (7.6) | 49.1 (11.7) |
| Time from diagnosis to transplantationT [months, *median(*(Q1;Q3*)*] | 33.1 (24.0;50.3) | 30.1 (21.9;50.3) | 33.1 (25.0;46.5) |
| Postoperative complications in the first 24 hours [n, %] | 18 (90.0) | 7 (87.5) | 11 (91.7) |
| Extracorporeal circulation need [n,(%)] | 4 (20.0) | 3 (37.5) | 1 (8.33) |
| Difficult weaning [n,*(%)*] (n=19) | 10 (52.6) | 3 (42.9) | 7 (58.3) |
| Tracheostomy [n,(%)] (n=19) | 10 (52.6) | 3 (42.9) | 7 (58.3) |
| Complications during the first month [n, %] (n=19) | 17 (89.5) | 6 (85.7) | 11 (91.7) |
| Complications after the first month [n, %] (n=19) | 18 (94.7) | 6 (85.7) | 12 (100.0) |
| >1 complication after transplantation [n, (%)]* (n=20) | 18 (90.0) | 6 (75.0) | 12 (100.0) |
| Allograft dysfunction[n, %] (n=19) | 8 (42.1) | 3 (42.9) | 5 (41.7) |
| Mechanical complications [n, (%)] (n=20) | 8 (40.0) | 3 (37.5) | 5 (41.7) |
| Nephrological complications [n, %] (n=19) | 7 (35.0) | 2 (25.0) | 5 (41.7) |
| Hematological complications [n, (%)] (n=20) | 6 (30.0) | 2 (25.0) | 4 (33.3) |
| Endocrine complications [n, (%)] (n=20) | 15 (75.0) | 5 (62.5) | 10 (83.3) |
| Infectious complications [n, (%)] (n=20) | 12 (60.0) | 3 (37.5) | 9 (75.0) |
| Positive pre-transplant CMV serology [n, %] | 18 (90.0) | 6 (75.0) | 12 (100) |
| Post-transplant CMV replication [n, %] (n=19) | 14 (73.7) | 5 (71.4) | 9 (75.0) |
| Hematological alterations due to CMV treatment [n, %] (n=15) | 4 (26.7) | 2 (40.0) | 2 (20.0) |
| CMV negativity after treatment [n, (%)] (n=16) | 9 (56.2) | 3 (60.0) | 6 (54.4) |
